# First-episode psychosis with comorbid cannabis use disorder: relapse prevention, prognosis, and mortality

**DOI:** 10.1192/j.eurpsy.2025.47

**Published:** 2025-08-26

**Authors:** S. Niemelä, Alexander Denissoff, Pihla Sassi, Antti Mustonen, Ellenor Mittendorfer-Rutz, Jari Tiihonen, Heidi Taipale

**Affiliations:** 1 Clinical Institute (Psychiatry), University of Turku; 2 Addiction Psychiatry Unit, Turku University Hospital, Turku, Finland

## Abstract

**Abstract: Introduction:**

Problematic cannabis use is prevalent among individuals with first-episode psychosis (FEP), with 35.6% meeting criteria for comorbid cannabis use disorder (CUD) (1). Cannabis use is associated with poorer FEP outcomes, including higher psychosis relapse risk (2). However, evidence on effective pharmacological treatments and long-term outcomes for FEP+CUD remains limited.

**Objectives:**

To evaluate outcomes of FEP with comorbid CUD and assess the real-world effectiveness of antipsychotic treatments in this population.

**Methods:**

This study analyzed a Swedish nationwide cohort using longitudinal register data (2006–2021). The sample included 1,820 patients with FEP+CUD (84.7% male, mean age 26.8 years). Outcomes included hospitalizations for psychotic relapse, any psychiatric disorder, or substance use disorder (SUD). Associations between antipsychotic use and outcomes were assessed using within-individual Cox regression models. Mortality rates were compared across FEP+CUD (n=2,154, mean age 25.0 years), cannabis-induced psychosis (CIP, n=1,263, mean age 25.0), and FEP without SUD (n=17,589, mean age 27.4), adjusting for age, sex, education, other SUDs, and disability pension status.

**Results:**

Over a mean follow-up of 6.13 years, 76% of participants were hospitalized for psychiatric diagnoses, 63% for SUD, and 61% for psychotic relapse. Antipsychotic use was associated with a 33% reduction in psychotic relapse risk. Clozapine and long-acting injectable (LAI) formulations of risperidone, aripiprazole, and paliperidone showed the greatest efficacy in relapse prevention. Clozapine reduced SUD-related hospitalizations by 86%. Within 10 years, 7.8% of FEP+CUD and 5.2% of CIP patients died, compared to 3.4% of FEP without SUD (adjusted odds ratio [aOR] 2.61, 95% CI 2.19–3.13, and aOR 1.67, 95% CI 1.28–2.17). Suicide was the leading cause of death in all groups, with higher rates in FEP+CUD (aOR 1.94, 95% CI 1.49–2.52) and CIP (aOR 1.70, 95% CI 1.21–2.37) compared to FEP without SUD.

**Conclusions:**

Clozapine and LAI formulations of second-generation antipsychotics (excluding olanzapine) or oral aripiprazole effectively prevent hospitalizations in FEP+CUD. Targeted efforts are needed to reduce premature mortality, particularly from suicide, in FEP patients with comorbid cannabis use disorder or cannabis-induced psychosis.

**References:**

1. Hunt GE, et al. Prevalence of comorbid substance use in schizophrenia spectrum disorders in community and clinical settings, 1990–2017: Systematic review and meta-analysis. Drug Alcohol Depend. 2018;191:234–258.

2. Bowtell M, et al. Clinical and demographic predictors of continuing remission or relapse following discontinuation of antipsychotic medication after a first episode of psychosis: A systematic review. Schizophr Res. 2018;197:9–18.

**Disclosure of Interest:**

S. Niemelä Consultant of: Recordati, Shire-Takeda, Speakers bureau of: Dne Pharma, Lundbeck, Recordati, Shire-Takeda

